# An Ultrasound Assessed Extraction Combined with Ion-Pair HPLC Method and Risk Assessment of Nitrite and Nitrate in Cured Meat

**DOI:** 10.1155/2018/1907151

**Published:** 2018-03-01

**Authors:** Babiker Yagoub Abdulkair, Amin O. Elzupir, Abdulaziz S. Alamer

**Affiliations:** ^1^Chemistry Department, College of Science, Al Imam Mohammad Ibn Saud Islamic University (IMSIU), Riyadh, Saudi Arabia; ^2^Ministry of Higher Education and Scientific Research, Chemistry Department, College of Science, Sudan University of Science and Technology (SUST), Khartoum, Sudan; ^3^Al Imam Mohammad Ibn Saud Islamic University (IMSIU), Committee on Radiation and Environmental Pollution Protection, Riyadh, Saudi Arabia; ^4^Ministry of Higher Education and Scientific Research, Central Laboratory, Sudan University of Science and Technology (SUST), Khartoum, Sudan

## Abstract

An accurate IPC-UV method was developed and validated for the determination of nitrite (NI) and nitrate (NA) in meat products. The best separation was achieved on a phenyl-hexyl column (150 mm × 4.6 mm, 3 *µ*m) with a mobile phase composed of 25% acetonitrile and 75% buffer (2 mM disodium hydrogen phosphate and 3 mM tetrabutylammonium bromide, pH = 4). Eluents were monitored at 205 nm. Linearity ranges were 1.86 × 10^−6^–7.5 *µ*g·ml^−1^ and 0.09–5.0 *µ*g·ml^−1^ for NI and NA, respectively. The correlation coefficients were greater than 0.999 for NI and NA. This method was applied to a number of processed meat products in Riyadh (*n* = 155). NI ranged from 1.78 to 129.69 mg·kg^−1^, and NA ranged from 0.76 to 96.64 mg·kg^−1^. Results showed extensive use of NI and NA; however, concentrations were within the legal limit of Saudi Arabia except for one sample. Further, the risk assessment and dietary exposure have been estimated for both NI and NA.

## 1. Introduction

Nitrite (NI) and nitrate (NA) are commonly used as food additives to cured meats as red color preservers, stabilizers, and antibacterials against *Clostridium botulinum*. However, in the stomach, at low pH, NI reacts with primary and secondary amines to form carcinogenic species known as nitrosamine compounds. In addition, as NA is readily reduced in vivo to NI by NA reductase, it can also be a source for nitrosamines. The data available from epidemiological studies show a positive correlation between NI and nitrosamine exposure and gastric cancer, oesophageal cancer, and leukemia. Some of these nitrosamines have been classified as group 1 carcinogens by the International Agency for Research on Cancer (IARC) [1–3].

NI and NA have been regulated by several bodies and organizations worldwide. The US Food and Drug Administration (FDA) has set 200 mg·kg^−1^ and 500 mg·kg^−1^ limits for NI and NA, respectively. A lower limit of 150 mg·kg^−1^ for both NI and NA has been set by the European Food Safety Authority (EFSA), while a 100 mg·kg^−1^ limit was set for NI in sterile meat products [4]. The Standardization Organization for the Gulf Cooperation Council (GSO) has set a 125 mg·kg^−1^ limit for both NI and NA in cured meat products [5].

For the measurement of NI, a Griess reaction is commonly used, which is based on diazotisation of NI using sulfanilamide and a subsequent reaction with *N*-(1-naphthyl) ethylenediamine. The yield of the colored product is quantified by spectrophotometry. To calculate the concentration of NA, it is reduced to NI. Then, the total NI concentration is measured, and the known NI concentration is subtracted from this total value. This reduction is carried out over a copper-activated cadmium reduction column. The disadvantage of this protocol is that it is a complicated process to get a complete reduction [6–8]. Instead, NI and NA can be separated and determined using ion chromatography (IC) with conductivity detection and ion-pair chromatography (IPC) with ultraviolet detection. The advantage of IPC over IC is that IC requires more analysis time, particularly when other anions, such as bromate, sulfate, and phosphate, are in the samples [8–17].

The Kingdom of Saudi Arabia (KSA) is one of the countries with the highest consumption of meat. A joint study from the Organization for Economic Co-operation and Development (OECD) and Food and Agriculture Organization (FAO) have reported that the meat consumption in the Kingdom of Saudi Arabia has increased to 50.8 kilograms per person per capita in 2015 [18]. Over the last two decades, a large number of meat factories have been deployed to meet the increasing demands. They provide this meat at prices much lower than fresh meat. However, there are no data about NI and NA in processed meat products consumed in KSA. The present research study has been conducted to determine the presence of nitrite and nitrate in processed meat in KSA.

## 2. Materials and Methods

### 2.1. Sampling

Sample collection was designed to represent the types of processed meat products consumed in KSA. Accordingly, 164 samples (400–2500 g) were collected from different supermarkets during June 2017 and kept at −20°C until analysis. The samples comprised 15 trademarks locally made by 19 different factories which could be divided into two groups: chicken products that included meatballs (7), kofta (8), nuggets (13), strips (11), kababs (6), hot dogs (14), minced meat (9), grilled chicken (8), and burgers (12) and red meat products that included meatballs (6), kofta (12), kababs (11), hot dogs (4), minced meat (20), and burgers (14).

### 2.2. Extraction Procedure

The samples were subsampled to about 20–50 g. Of these, 5 g was weighed and placed in a 100 ml beaker. Then, 30 ml Millipore water and 3 ml of saturated borax solution were added. The mixture was placed in an ultrasonic bath and sonicated for 15 min at 40°C. Then, 2 ml potassium ferrocyanide solution (0.25 M) was added to the extract, which was mixed and left for 5 min. 10 ml of the extract was centrifuged at 6000 rpm for 5 min, and 1 ml was taken from the supernatant for HPLC analysis.

### 2.3. Separation and Quantification

Separation of NI and NA was performed by high-performance liquid chromatography (HPLC) with UV-Vis detection using an HPLC apparatus (PerkinElmer, USA) consisting of a UV-Vis detector, binary analytical pump, vacuum degasser, and autosampler equipped with a 100 *µ*l loop. The chromatographic column used was an RP-thermophenyl hexyl column (150 mm × 4.6 mm with 3 *µ*m particles). The wavelength was set at 205 nm. Isocratic elution with a flow rate of 0.7 ml·min^−1^ was used. The mobile phase was composed of 25% acetonitrile and 75% buffer (2 mM disodium hydrogen phosphate and 3 mM tetrabutylammonium bromide, adjusted to pH 4 by phosphoric acid). Standard concentrations of NI at 0.019, 0.056, 0.20, 2.0, 10.0, 20.0, 40.0, and 100.0 *µ*g·ml^−1^ and of NA at 0.63, 0.1, 1.0, 5.0, 10.0, 20.0, and 50.0 *µ*g·ml^−1^ were prepared to check for linearity and quantify the analysed samples. LOD and LOQ were calculated from linearity data as per the USP protocol for method validation 2017 as follows:

(1) $$\begin{array}{c} \text{LOD}=3*\frac{\text{SD}}{\text{slope}},\\ \text{LOQ}=10*\frac{\text{SD}}{\text{slope}}. \end{array}$$

### 2.4. Recovery Study

The accuracy of the extraction method was tested by its recovery. Preanalysed minced fresh meat samples were spiked with concentrations of 50, 100, 150, and 200 *µ*g·g^−1^ of both NI and NA. For repeatability purposes, the extraction was performed three times for each concentration. Each extract was analysed in triplicate by HPLC.

### 2.5. Dietary Exposure and Risk Assessment

The average consumption of processed red meat was reported to be 18 g per person per day and 129 g for the corresponding poultry meat as documented by the United States Department of Agriculture [19]. The average body weight per person was assumed to be 60 kg. The estimated daily intake (EDI) of NI and NA in processed meat was calculated as below:

(2) $$\begin{array}{c} \text{dietary exposure}=\frac{\text{mean consumption}\left(g\right)\times \text{concentration}\left(\text{mg}/\text{kg}\right)}{\text{body weight}\left(\text{kg}\right)\times 1000}. \end{array}$$

The health risk was assessed using the margin of exposure (MOE) approach. The MOE is calculated as the ratio between the benchmark dose lower confidence limit (BMDL_10_) that causes a 10% increase in tumour cell formation in rat and the estimated average consumption of nitrite in processed meat [20]. Nitrosodiethylamine (NDEA) and nitrosodimethylamine (NDMA) have been suggested to represent the most potent nitroso compounds. NDEA and NDMA have been exhibiting BMLD_10_ of 18 and 27 *μ*g/kg body weight/day, respectively [21]:

(3) $$\begin{array}{c} \text{MOE}=\frac{{\text{BMDL}}_{10}}{\text{dietary exposure}}. \end{array}$$

## 3. Results and Discussion

### 3.1. Validation of Extraction and Assay Method

An IPC-UV method was developed and validated for determination of NI and NA in processed meat products. In order to obtain an excellent separation, several solvents were tried with different salts and different percentages. The best separation occurred using acetonitrile and buffer solution at a ratio of 1 : 3. Increasing the buffer led to better resolution but reduced NI detection sensitivity, and vice versa. The linearity, limit of detection, limit of quantification, and accuracy were examined (Tables 1 and 2). NI and NA are added to meat in the form of sodium and potassium salts, which are soluble in water, so they are generally extracted by hot water and boiling [22]. However, NI is known to be unstable and can quickly turn into nitrates and/or nitric oxide at high temperatures [11]. Because of the high efficiency of ultrasonic baths to enhance solubility, one of them was used for extraction, instead of boiling. The use of ultrasonic bath led to high recoveries of 96–101% for NI and 96–100% for NA. The accuracy and sensitivity of the method were considered sufficient for NI and NA analysis in meat products (Table 2; see supplementary materials for more details (available here)). To our knowledge, this is the first method of extracting nitrite and nitrate simultaneously from the processed meat products using ultrasonic technique. Further, the extraction was performed at low temperature (40°C), thus reducing the likelihood of nitrite oxidation. Furthermore, recovery was found to be high (approximately 100%).

### 3.2. Nitrite and Nitrate in Processed Meat Products

Nitrite was detected in all samples (*n* = 155), with a concentration range of 1.78 to 129.69 mg·kg^−1^ and a mean of 31.71 ± 23.83 mg·kg^−1^. NA was detected in 120 samples (75%), with a concentration range of 0.76–96.64 mg·kg^−1^ and a mean of 13.47 ± 13.76 mg·kg^−1^. For specific categories, NI in red meat ranged from 3.03 to 112.71 mg·kg^−1^ with a mean of 31.67 ± 22.22 mg·kg^−1^, while NA ranged from 0.76 to 96.64 mg·kg^−1^ with a mean of 15.90 ± 15.89. In chicken products, NI ranged from 1.78 to 129.96 mg·kg^−1^ with a mean of 31.75 ± 25.11 mg·kg^−1^, while NA ranged from 0.78 to 51.34 mg·kg^−1^ with a mean of 12.13 ± 11.61 mg·kg^−1^, as shown in Table 3.

Our results showed that NI and NA concentration varied according to the meat products. The highest level of NI was found in chicken meatballs followed by chicken kabab, red meat kofta, and red meat burgers. In the same manner, NA was highest in red meat hot dogs, followed by chicken kababs, red meat meatballs, chicken hot dogs, and red meat burgers. NI and NA were found in minced meat at low levels, which may be due to the product containing a large percentage of soybeans or peanuts. The legal limit of NI and NA in processed meat products is 125 mg·kg^−1^ according to GSO, the adopted regulation in the KSA [5]. However, 99.35% (*n* = 154) of the analysed samples were within the legal limit, while 0.65% (*n* = 1) was found to exceed the limit. Although most products contained NI and NA within the permissible limits, when taken in large quantities, exposure may be increased, which may contribute to an increased risk of stomach cancer.

Given the seriousness of the presence of NI and NA in meat, a number of studies have been conducted to determine their quantities and compliance with the permissible limit. Compared to those studies, the range of NI in the present study was higher than that reported in Australia (3.7–86.7 mg·kg^−1^), Turkey (0.17–2.33 mg·kg^−1^ and 3.288–2.622 mg·kg^−1^), and Sudan (28.0–55.7 mg·kg^−1^), and lower that those reported in USA (0.0–36.5 mg·kg), Denmark (60–150 mg·kg^−1^), Iran (96–168 mg·kg^−1^), Korea (0.0–60 mg·kg), and China (0.93–158 mg·kg^−1^). On the other hand, the range of NA was lower than those reported in Australia (3.7–139.5 mg·kg^−1^), Turkey (83.14–150.89 mg·kg^−1^, 53–400 mg·kg^−1^, and 12–555 mg·kg^−1^), Denmark (5.3–230 mg·kg^−1^), and Iran (167–763 mg·kg^−1^) [11, 19, 27, 28–30].

### 3.3. Dietary Exposure and Risk Assessment

The averages of the estimated daily intake (EDI) of NI and NA are shown in Table 4. The EDI for the majority of the analysed samples was found to be within the accepted daily intake (ADI) of 0–0.07 and 0–3.7 mg/kg body weight/day for NI and NA, respectively, which was adopted by Scientific Committee on Food (SCF) and JECFA [31]. Otherwise, the average concentration of NI in chicken meatball samples is more than the ADI and hence may pose a risk to the average of the consumers.

On the other hand, the MOE was also calculated for the analysed samples as shown in Table 4. The MOE is a newly developed approach for measuring the risk of carcinogens, recommended by the European Food Safety Authority (EFSA) and the Joint FAO/WHO Expert Committee on Food Additives (JECFA) [32]. The MOE was estimated for NI and NA, using response modelling set by the Scientific Committee on Consumer Safety (SCCS) of the European Commission for NDEA and NDMA, representing the most potent nitroso compounds. The MOE equal or greater than 10,000 would be of low concern, and vice versa [21]. Our results revealed that the MOE was found to be ranged from 147 to 946 for NI in poultry meat and from 2237 to 7728 for red meat and ranged from 387 to 7914 for NA in poultry meat and from 2568 to 13,027 for red meat. Unexpectedly, processed red meat products are safer than poultry products in terms of the presence of NI and NA; this is because NI and NA are used to keep meat reddening. However, NI and NA may be added in poultry meat products, with a high concentration, because they are needier than red meat to develop their taste.

Finally, NI and NA salts are used in cured meat in order to enhance textures and flavors for both poultry and red meat products, to preserve the unique color of red meat, and to prevent lipid oxidation. Now, there are many alternatives which can be used instead of NI with less potential health risk such as green tea extract, rosemary and oregano extracts, annatto (*Bixa orellana* L.), grape seed extract, pine bark extract, and rosemary oleoresin. However, even with all these alternatives and the health risk attributed to the use of NI, it is still commonplace in meat manufacturing [1]. The challenge remains for regulators to apply these or other alternative preservatives, which can reduce the cancer risks associated with exposure to NI and NA salts.

## 4. Conclusion

To conclude, the use of NI and NA in meat as a preservative is of great interest, with consequent health risks, for the formation of carcinogenic nitroso compounds. In the present study, an accurate and precise IPC-UV method for determination of NI and NA was developed and validated. The incidences of NI and NA in meat products in Riyadh, KSA, were tested, and the majority of the samples were found to be within the permissible limit for both NI and NA, according to GSO. However, the dietary exposure to NI in some chicken meatball samples exceeds the ADI. Further, the presence of NI and NA in these products in low concentration may still pose a risk due to chronic exposure. In general, poultry products have been found to contain higher concentrations of nitrite than that of red meat products. To the best of our knowledge, this is the first report about NI and NA in processed meat products in the Kingdom of Saudi Arabia. It has been recommended to analyse NI and NA in vegetables and fruits and to expand this work to cover other areas in the KSA.

## Figures and Tables

**Table 1:** Quality parameters and method performance.

Parameters	Sodium nitrite	Sodium nitrate
LOD (ng·ml^−1^)	0.0006	27.4
LOQ (ng·ml^−1^)	0.002	91.4
Correlation coefficient	0.9997	0.9996
Linearity range (*µ*g·ml^−1^)	1.86 × 10^−6^–7.5	0.09–5.0

LOD = limit of detection; LOQ = limit of quantification.

**Table 2:** Recovery rates from meat spiked with different concentrations of nitrite and nitrate.

Sodium nitrite			Sodium nitrate		
Spiked level (*µ*g·g^−1^)	Recovery (%)	SD	Spiked level (*µ*g·g^−1^)	Recovery (%)	SD
50	96	13.68	50	96	7.13
100	100	9.64	100	96	16.65
150	98	9.12	150	100	3.28
200	101	3.19	200	98	6.76

SD = standard deviation.

**Table 3:** Nitrite and nitrate in processed meat products in Riyadh, KSA.

Category	Meat product	Number of samples	Sodium nitrite (mg·kg^−1^)		Sodium nitrate (mg·kg^−1^)	
			Range	Average ± SD	Range	Average ± SD
Chicken	Meatball	7	24.49–129.69	84.92 ± 41.40	7.06–18.15	12.69 ± 4.87
	Kofta	8	15.63–49.36	33.47 ± 13.10	2.59–29.37	11.14 ± 10.88
	Nuggets	13	1.78–49.87	24.26 ± 13.35	1.37–12.84	6.49 ± 3.75
	Strips	11	8.62–42.66	19.91 ± 11.46	0.78–8.72	2.15 ± 2.50
	Kabab	6	6.96–73.92	41.08 ± 27.51	10.98–73.95	29.30 ± 22.80
	Hotdog	14	5.39–91.74	22.62 ± 22.40	5.35–50.65	25.49 ± 13.16
	Minced meat	9	9.18–36.20	20.27 ± 8.74	1.83–8.59	3.87 ± 2.53
	Chicken grill	8	6.11–67.06	33.47 ± 18.38	4.53–26.72	13.29 ± 8.21
	Burger	12	11.60–54.95	32.03 ± 12.23	1.69–10.75	6.68 ± 3.15

Red meat	Meatball	6	8.28–46.67	27.97 ± 14.77	18.66–35.66	27.29 ± 7.36
	Kofta	12	5.95–73.15	40.23 ± 20.99	4.33–26.50	12.22 ± 6.81
	Kabab	11	6.46–52.42	25.85 ± 15.88	3.86–96.64	23.96 ± 28.17
	Hotdog	4	9.31–83.10	17.47 ± 13.86	30.30–34.31	31.65 ± 2.30
	Minced meat	20	3.03–70.87	27.82 ± 16.19	0.76–17.13	9.36 ± 4.43
	Burger	14	4.76–112.71	40.03 ± 34.16	1.45–89.31	15.00 ± 23.48

SD = standard deviation.

**Table 4:** Estimated daily intake (EDI; mg/kg BW/day) for nitrite and nitrate ions, and margin of exposure based on nitrosodiethylamine (NDEA) and nitrosodimethylamine (NDMA) for nitrite and nitrate in processed meat products commercialized in Riyadh, KSA.

Category	Meat product	Nitrite			Nitrate		
		EDI	MOE		EDI	MOE	
			NDEA	NDMA		NDEA	NDMA
Chicken meat	Meatball	0.122	148	222	0.020	894	1341
	Kofta	0.048	375	563	0.018	1018	1527
	Nuggets	0.035	518	776	0.010	1748	2622
	Strips	0.029	631	946	0.003	5276	7914
	Kabab	0.059	306	459	0.046	387	581
	Hotdog	0.032	555	833	0.040	445	667
	Minced meat	0.029	620	929	0.006	2931	4396
	Chicken grill	0.048	375	563	0.021	853	1280
	Burger	0.046	392	588	0.011	1698	2547

Red meat	Meatball	0.006	3218	4827	0.006	2979	4468
	Kofta	0.008	2237	3356	0.003	6652	9978
	Kabab	0.005	3482	5222	0.005	3393	5089
	Hotdog	0.003	5152	7728	0.007	2568	3853
	Minced meat	0.006	3235	4853	0.002	8685	13,027
	Burger	0.008	2248	3372	0.003	5419	8129

MOE = margin of exposure; EDI = estimated daily intake (mg/kg BW/d for nitrite ion); NDEA = nitrosodiethylamine; NDMA = nitrosodimethylamine.

## Supplementary Materials

Supplementary Materials The chromatograms of separated nitrite and nitrate standards, as well as that in different types of processed meat products, have been shown in supplementary materials.

## References

[1] Alahakoon A. U., Jayasena D. D., Ramachandra S., Jo C. (2015). Alternatives to nitrite in processed meat: up to date. *Trends in Food Science & Technology*.

[2] Sindelar J. J., Milkowski A. L. (2011). Sodium nitrite in processed meat and poultry meats: a review of curing and examining the risk/benefit of its use. *American Meat Science Association White Paper Series*.

[3] Qiu Y., Chen J. H., Yu W., Wang P., Rong M., Deng H. (2017). Contamination of Chinese salted fish with volatile N-nitrosamines as determined by QuEChERS and gas chromatography-tandem mass spectrometry. *Food Chemistry*.

[4] Food Standards Agency, UK (2015). Food additives legislation guidance to compliance, food additives–regulation 1333/2008. https://www.food.gov.uk/sites/default/files/multimedia/pdfs/guidance/food-additives-legislation-guidance-to-compliance.pdf.

[5] Standardization Organization for Gulf Cooperation Council G.C.C (2012). Chilled Marinated Meats. https://members.wto.org/crnattachments/2013/tbt/KWT/13_0480_00_x.pdf.

[6] Tsikas D. (2007). Analysis of nitrite and nitrate in biological fluids by assays based on the Griess reaction: appraisal of the Griess reaction in the L-arginine/nitric oxide area of research. *Journal of Chromatography B*.

[7] Masserini R. T., Fanning K. A. (2000). A sensor package for the simultaneous determination of nanomolar concentrations of nitrite, nitrate, and ammonia in seawater by fluorescence detection. *Marine Chemistry*.

[8] Zuo Y., Wang C., Van T. (2006). Simultaneous determination of nitrite and nitrate in dew, rain, snow and lake water samples by ion-pair high-performance liquid chromatography. *Talanta*.

[9] Hai-ying B., Xiao-ming L. I., Fei W. (2013). Determination of bromate nitrite and nitrate in foods by HPLC. *Food Research and Development*.

[10] Croitoru M. D. (2012). Nitrite and nitrate can be accurately measured in samples of vegetal and animal origin using an HPLC-UV/VIS technique. *Journal of Chromatography B*.

[11] Hsu J., Arcot J., Lee N. A. (2009). Nitrate and nitrite quantification from cured meat and vegetables and their estimated dietary intake in Australians. *Food Chemistry*.

[12] Helaleh M. I., Korenaga T. (2000). Ion chromatographic method for simultaneous determination of nitrate and nitrite in human saliva. *Journal of Chromatography B: Biomedical Sciences and Applications*.

[13] Jedlickova V., Paluch Z., Alušík Š. (2002). Determination of nitrate and nitrite by high-performance liquid chromatography in human plasma. *Journal of Chromatography B*.

[14] Lopez-Moreno C., Perez I. V., Urbano A. M. (2016). Development and validation of an ionic chromatography method for the determination of nitrate, nitrite and chloride in meat. *Food Chemistry*.

[15] Vahid S., Dashti-Khavidaki S., Sormaghi M. S., Ahmadi F., Amini M. (2012). A new pre-column derivatization method for determination of nitrite and nitrate in human plasma by HPLC. *Journal of Liquid Chromatography & Related Technologies*.

[16] Wu F. Z., Wang H. (2010). Determination of nitrite and nitrate in vegetables, fruits and derived products by HPLC. *Chinese Journal of Health Laboratory Technology*.

[17] Zeng X. L., Ye M. L., Chen Y. X., Zhu Y. (2006). Accelerated solvent extraction-ion chromatography with conductivity detection for the extraction and determination of nitrate and nitrite in meat. *Journal of Instrumental Analysis*.

[18] The Joint of the Organization for Economic Co-Operation and Development (OECD) and Food and Agriculture Organization (FAO) (2016). Meat consumption, beef and veal/pork meat/poultry meat/sheep meat, kilograms/capita, 2015. https://data.oecd.org/agroutput/meat-consumption.htm.

[19] United States Department of Agriculture (USDA) (2017). *Saudi Arabia Milled Rice Domestic Consumption by Year*.

[20] WHO/IPCS (2009). *Environmental Health Criteria 239: Principles for modelling dose–response for the risk assessment of chemicals*.

[21] Habermeyer M., Roth A., Guth S. (2015). Nitrate and nitrite in the diet: how to assess their benefit and risk for human health. *Molecular Nutrition & Food Research*.

[22] Adam A. H. B., Mustafa N. E. M., Rietjens I. M. (2017). Nitrite in processed meat products in Khartoum, Sudan and dietary intake. *Food Additives & Contaminants: Part B*.

[23] Choi S. H., Suh H. J. (2017). Determination and estimation of daily nitrite intake from processed meats in Korea. *Journal of Consumer Protection and Food Safety*.

[24] Nunez De González M. T., Osburn W. N., Hardin M. D. (2012). Survey of residual nitrite and nitrate in conventional and organic/natural/uncured/indirectly cured meats available at retail in the United States. *Journal of Agricultural and Food Chemistry*.

[25] Erkekoglu P., Sipahi H., Baydar T. (2006). Nitrite residues of bouillons marketed in Turkey. *FABAD Journal of Pharmaceutical Sciences*.

[26] Erkekoglu P., Sipahi H., Baydar T. (2009). Evaluation of nitrite in ready-made soups. *Food Analytical Methods*.

[27] Gao Q., Feng W., Chang Z. (2011). Risk monitoring of nitrite content in some cooked meat products in Tianjin. *Occupation and Health*.

[28] Leth T., Fagt S., Nielsen S., Andersen R. (2008). Nitrite and nitrate content in meat products and estimated intake in Denmark from 1998 to 2006. *Food Additives & Contaminants: Part A, Chemistry, Analysis, Control, Exposure & Risk Assessment*.

[29] Sadeghi E., Hashemian A. H., Soltanian M., Soltanian S., Mohammadi M. (2014). Study of nitrite and nitrate levels in meat products distributed in Kermanshah. *Iran Occupational Health*.

[30] Sungur Ş., Atan M. M. (2013). Determination of nitrate, nitrite and perchlorate anions in meat, milk and their products consumed in Hatay region in Turkey. *Food Additives and Contaminants: Part B*.

[31] FAO/WHO (2003). Nitrite and potential endogenous formation of N-nitroso compounds. *WHO Food Additive series*.

[32] European Food Safety Authority (EFSA) (2005). Opinion of the scientific committee on a request from EFSA related to a harmonized approach for risk assessment of substances which are both genotoxic and carcinogenic. *EFSA Journal*.

