# Supplementary material for: An Ultrasound Assessed Extraction Combined with Ion-Pair HPLC Method and Risk Assessment of Nitrite and Nitrate in Cured Meat
Source: J Anal Methods Chem. 2018 Mar 1;2018:1907151. doi: 10.1155/2018/1907151 (PMC5852839; doi:10.1155/2018/1907151)
Supplement: Supplementary Materials — The chromatograms of separated nitrite and nitrate standards, as well as that in different types of processed meat products, have been shown in supplementary materials. [file 1907151.f1.docx]

**Supplementary materials**

**Dear Editor,**

**Herein some selected chromatograms for review.**

**With the best regards,**

Chromatogram 1: Nitrite and Nitrate standard separated by optimized IPC condition (See materials and methods section)

Chromatogram 2: Nitrite and Nitrate free from minced meat prepared in home, as blank for recovery studies.

Chromatogram 3: blank spiked with Nitrite and Nitrate.

Chromatogram 4: Nitrite and Nitrate in **meatballs** sample (NI = 25.12 mg Kg^-1^, NA= 24.4 mg Kg^-1^).

Chromatogram 5: Nitrite and Nitrate in **meatballs** sample (NI = 46.67 mg Kg^-1^, NA= 30.42 mg Kg^-1^).

**Chromatogram 6: Nitrite in burger sample (NI = 81.94 mg Kg^-1^).**

**Chromatogram 7: Nitrite in burger sample (NI = 20.16 mg Kg^-1^).**

**Chromatogram 8: Nitrite and Nitrate in chicken grill sample (NI = 45.21 mg Kg^-1^, NA= 26.72 mg Kg^-1^).**

**Chromatogram 9: Nitrite and Nitrate in minced meat sample (NI = 23.06 mg Kg^-1^, NA= 8.59 mg Kg^-1^).**

**Chromatogram 10: Nitrite and Nitrate in minced meat sample (NI = 70.87 mg Kg^-1^, NA= 11.03 mg Kg^-1^).**

**Chromatogram 11: Nitrite in kofta sample (NI = 48.94 mg Kg^-1^).**

**Chromatogram 12: Nitrite and Nitrate in kabab** **sample (NI = 52.42 mg Kg^-1^, NA= 96.64 mg Kg^-1^).**

**Chromatogram 13: Nitrite in Strips sample (NI = 18.24 mg Kg^-1^).**

Chromatogram 14: Nitrite and Nitrate detected in **minced meat** sample (NI = , NA=	)……. Smp75

Chromatogram 15: Nitrite and Nitrate detected in **minced meat** sample (NI = , NA=	)……. Smp85
